# Measuring causes of death in populations: a new metric that corrects cause-specific mortality fractions for chance

**DOI:** 10.1186/s12963-015-0061-1

**Published:** 2015-10-12

**Authors:** Abraham D. Flaxman, Peter T. Serina, Bernardo Hernandez, Christopher J. L. Murray, Ian Riley, Alan D. Lopez

**Affiliations:** Institute for Health Metrics and Evaluation, University of Washington, 2301 Fifth Ave., Suite 600, Seattle, WA 98121 USA; University of Queensland, School of Population Health, Level 2 Public Health Building School of Population Health, Herston Road, Herston, QLD 4006 Australia; University of Melbourne School of Population and Global Health Building 379, 207 Bouverie St, Parkville, 3010 VIC Australia

## Abstract

**Background:**

Verbal autopsy is gaining increasing acceptance as a method for determining the underlying cause of death when the cause of death given on death certificates is unavailable or unreliable, and there are now a number of alternative approaches for mapping from verbal autopsy interviews to the underlying cause of death. For public health applications, the population-level aggregates of the underlying causes are of primary interest, expressed as the cause-specific mortality fractions (CSMFs) for a mutually exclusive, collectively exhaustive cause list. Until now, CSMF Accuracy is the primary metric that has been used for measuring the quality of CSMF estimation methods. Although it allows for relative comparisons of alternative methods, CSMF Accuracy provides misleading numbers in absolute terms, because even random allocation of underlying causes yields relatively high CSMF accuracy. Therefore, the objective of this study was to develop and test a measure of CSMF that corrects this problem.

**Methods:**

We developed a baseline approach of random allocation and measured its performance analytically and through Monte Carlo simulation. We used this to develop a new metric of population-level estimation accuracy, the Chance Corrected CSMF Accuracy (CCCSMF Accuracy), which has value near zero for random guessing, and negative quality values for estimation methods that are worse than random at the population level.

**Results:**

The CCCSMF Accuracy formula was found to be CCSMF Accuracy = (CSMF Accuracy - 0.632) / (1 - 0.632), which indicates that, at the population-level, some existing and commonly used VA methods perform worse than random guessing.

**Conclusions:**

CCCSMF Accuracy should be used instead of CSMF Accuracy when assessing VA estimation methods because it provides a more easily interpreted measure of the quality of population-level estimates.

**Electronic supplementary material:**

The online version of this article (doi:10.1186/s12963-015-0061-1) contains supplementary material, which is available to authorized users.

## Introduction

Understanding the leading cause of death (CoD) is vital information for health decision-making [1]. The civil and vital registration system (CVRS) constitutes the most timely and accurate source of this information [2, 3], but is unavailable in many regions of the world [4]. Verbal autopsy interviews (VAIs) provide a promising alternative (and potentially a complement) to the CVRS approach in settings where CVRS information is unavailable or unreliable [5, 6]. In populations where medical certification of causes of death is difficult to achieve, particularly those poorly serviced by health facilities, the only viable option to obtain information on causes of death is to use verbal autopsy (VA) methods. VA includes three components: (1) a VA instrument, used to elicit information from the family or relatives about signs and symptoms experienced by the deceased prior to death; (2) a diagnostic method to derive the most probable cause of death from these responses to the VA interview with families, which has traditionally been accomplished by physician review, but can also be assessed using a diagnostic algorithm that recognizes and associates response patterns with likely causes of death; and (3) a target cause of death list that covers the universe of causes of death which can be diagnosed from the VA interview, irrespective of the diagnostic approach followed. Worldwide, less than 40 % of deaths are medically certified each year [7], and an additional 100,000 or so are currently assigned a cause by some variant of verbal autopsy, mostly in routine mortality surveillance systems operating in China [8], India [9], or the INDEPTH network [10]. There is now increasing momentum worldwide to apply cost-effective VA methods to facilitate the introduction of VA into routine civil registration systems in countries across Asia, Africa and Latin America [11].

It is technically challenging to predict the underlying cause of death from VAIs. A recent paper compared the quality of six prediction methods on VAIs where the underlying cause was known to meet rigorous clinical diagnostic criteria [12]. In that work, prediction quality was assessed with five different metrics. Most of these measure predictive quality on the individual level, to quantify how well a method predicts the cause of each death. However, for public health policy, it is of great importance to make accurate predictions at the population level. Cause-specific mortality fraction (CSMF) accuracy is a recently developed metric for quantifying prediction quality at the population level [13]. CSMF accuracy is an index of absolute deviation of a set of estimated CSMFs from the true CSMF distribution, with value of one meaning perfect agreement, and value of zero meaning as far apart as possible. This metric is specific to validation studies which make use of a database of VAIs with known underlying cause of death (labeled data, in the parlance of machine learning). To protect against “over-fitting”, (where an algorithm, or even a physician coder, estimates a CSMF distribution based on what they have seen in the past instead of the data that they are currently examining), CSMF accuracy requires repeated calculation of this absolute deviation index for multiple random samples of the underlying cause distribution of the test data.

CSMF accuracy, as originally formulated, is misleading, however. It is always a value that falls between zero and one, but in practice it is rarely lower than 0.5 [12]. This is a limitation in interpretation, because even a very low-quality approach scores well above zero. An extreme example is that of a “prediction” method that resorts simply to random guessing. Even this information-free approach yields a CSMF accuracy substantially above zero.

In this paper, we propose a modification of the CSMF Accuracy metric, which we call chance-corrected cause-specific mortality fraction (CCCSMF) accuracy, to adjust the baseline of the metric so that allocating causes uniformly at random (i.e. just “by chance”) achieves an expected accuracy score of zero. We believe that this will improve the interpretation of the absolute and comparative performance of different methods for estimating cause-of-death patterns in populations.

The remainder of the paper is organized as follows: in the methods section, we define the baseline algorithm of Random Allocation and review the definitions of chance-corrected concordance and CSMF accuracy. We then introduce the Random-From-Train algorithm and reiterate the importance of randomly resampling the distribution of the test set when the population-level predictions are of primary interest, and define our new metric of CCCSMF accuracy. In the results section, we use Monte Carlo simulation to find the exact formula of CCCSMF accuracy, and to replicate the analytically derived chance-corrected concordance metric. We then apply this formula to produce a plot comparing three existing methods of coding VAIs in terms of CCC and CCCSMF accuracy, which we view as chance-correcting previous results. We conclude the results section with a demonstration of the importance of randomly resampling the distribution of the test set. We follow this with discussion and conclusions sections, including a subsection discussing the limitations of our work.

## Methods

In Machine Learning (ML), mapping from VAIs to CoD is an example of a classification problem, and ML methods for classification, such as neural networks [14], *k-*nearest neighbor [15], and random forests [16] have been applied to VAIs previously. An ML concept that is also quite relevant for VA applications is that of the “baseline approach”, where a simple approach is used as a comparison for the more sophisticated classification methods. A baseline approach for mapping VAIs to CoD is *Random Allocation*, which allocates the cause of death uniformly at random from a mutually exclusive, collectively exhaustive cause list (Table 1).

**Table 1:** Random allocation algorithm

Training Data: mutually exclusive, collectively exhaustive cause list (*C*_1_, *C*_2_, …, *C*_*J*_), fixed a priori.
Input: VAI results *X*.
Output: Cause C, selected uniformly at random from (*C*_1_, *C*_2_, …, *C*_*J*_).

The machine learning construct of the *confusion matrix* is a useful tool for understanding the performance of a classifier on labeled validation data. The confusion matrix *M* is a cross-tabulation of the number of true and predicted cases for each cause, which is to say a *J* × *J* matrix where *J* is the length of the mutually exclusive, collectively exhaustive cause list with the entry in row *j* and columns *j*' given by

$$ {M}_{j,{j}^{\prime }}=\#\  of\  VAIs\  with\  true\  CoD\ j\  and\  predicted\  CoD\ {j}^{\prime } $$

Table 2 shows the confusion matrix for physician-coded VA and random allocation for the Population Health Metrics Research Consortium (PHMRC) validation database for gold-standard-level-one adult deaths (this database consisted of 2,702 VAIs gathered from six sites in four countries, for deaths that met stringent clinical diagnostic criteria [17], and were subsequently coded by physicians in a validation of the PCVA approach to VA [18]).

**Table 2:** Confusion matrices for physician-certified verbal autopsy and random-allocation verbal autopsy.

a) Physician Certified VA Confusion Matrix					b) Random Allocation Confusion Matrix				
		Predicted					Predicted		
		Stroke	Diabetes	Other			Stroke	Diabetes	Other
True	Stroke	123	18	125	True	Stroke	87	84	95
	Diabetes	5	86	55		Diabetes	32	61	53
	Other	83	95	2112		Other	746	780	764

Panel A shows the confusion matrix for physician certified verbal autopsy (with a length-three cause list for clarity). The entry in each cell counts the number of deaths truly due to the row cause that were predicted to be due to the column cause. For example, the value 83 in the “other” row, “stroke” column indicates that 83 deaths truly due to causes other than stroke or diabetes were (incorrectly) attributed to stroke by physicians. This table demonstrates that (for this dataset) physicians are right more often than they are wrong when they predict stroke as the cause of death, but wrong more than they are right when they predict diabetes. Panel B shows the confusion matrix for Random Allocation with the same dataset, where random chance predicts stroke and diabetes incorrectly for a vast majority of the cases. True and PCVA data from Lozano et al. [18, 22], where physicians were presented with VAI data where the underlying cause was known to meet stringent clinical diagnostic criteria, and their results compared to the truth

### Chance-correcting concordance and CSMF accuracy

Recent work by Murray et al. developed robust metrics for individual-level and population-level prediction quality: chance-corrected concordance and CSMF accuracy [13]. Both can be written easily in terms of the confusion matrix. Cause-specific concordance (*C*_*j*_) is a measure of predictive quality at the individual level, which quantifies how likely a prediction is to be correct for a single VAI. It is equal to the fraction of VAIs where the prediction was correct, or in other words,

$$ {C}_j=\frac{M_{j,j}}{{\displaystyle {\sum}_{j^{\prime }=1}^J}{M}_{j,{j}^{\prime }}}. $$

Then cause-specific chance-corrected concordance (CCC_*j*_) has the form

$$ {\mathrm{CCC}}_j = \frac{C_j-1/J}{1 - 1/J}. $$

This scales and shifts the concordance so that the expected CCC_*j*_ of random allocation is zero. Finally, an overall metric of chance-corrected concordance is calculated as an unweighted mean of the cause-specific values:

$$ \mathrm{C}\mathrm{C}\mathrm{C} = \frac{1}{J}{\displaystyle \sum_{j=1}^J}{\mathrm{CCC}}_j. $$

Chance-corrected concordance is an adaptation of a generalization of the sensitivity metric so familiar in epidemiology. It is generalized to account for the polytomous nature of the prediction task. The chance correction is important for making comparisons between classifiers designed for different-length cause lists—shortening the cause list makes the concordance of Random Allocation go up, but leaves CCC unchanged at zero.

CSMF accuracy is a measure of predictive quality at the population level, which quantifies how closely the estimated CSMF values approximate the truth. It can be defined in terms of the normalized row and column sums of the confusion matrix, ${\mathrm{CSMF}}_j^{\mathrm{true}}={\sum}_{j^{\prime }=1}^J{M}_{j,{j}^{\prime }}/\mathrm{n}$, and ${\mathrm{CSMF}}_j^{\mathrm{pred}}={\sum}_{j^{\prime }=1}^J{M}_{j^{\prime },j}/\mathrm{n}$, where *n* is the total number of VAIs,

$$ n=\sum_{j=1}^J\sum_{j^{\prime }=1}^J{M}_{j,{j}^{\prime }}. $$

In this notation,

$$ \mathrm{CSMF}\ \mathrm{accuracy}=1 - \frac{{\displaystyle {\sum}_{j=1}^J}\left|{\mathrm{CSMF}}_j^{\mathrm{true}}-{\mathrm{CSMF}}_j^{\mathrm{pred}}\right|}{2\left(1-{ \min}_j\left({\mathrm{CSMF}}_j^{\mathrm{true}}\right)\right)}, $$

which has minimum value zero and maximum value one. Unlike CCC, the CSMF accuracy of Random Allocation is greater than zero, a deficiency that this paper seeks to remedy.

### The importance of randomly resampling the CSMF distribution

These metrics have been widely used in measuring and comparing the quality of a range of verbal autopsy analysis methods [16, 18–22] and their use is complicated by the need to consider the average CCC and CSMF over the range of possible CSMF distributions. This is particularly relevant for CSMF accuracy, because a classifier that knows the CSMF distribution a priori could perform very well at the population level for that CSMF distribution without getting anything right at the individual level. This might seem like a purely theoretical concern, but a recent paper comparing four approaches to computer certified verbal autopsy methods omitted CSMF distribution resampling, and which led to reporting counter-intuitive and misleading results [23]. To demonstrate this in an extreme example, we developed the population-level prediction scheme *Random-From-Train*, where the prediction is random, but with a distribution derived from the training dataset (hence the name Random-From-Train). This is subtly different from the distribution used in the Random Allocation predictor, and designed so that, in expectation, the CSMFs predicted for the test set match the CSMFs observed in the training set (Table 3).

**Table 3:** Random-From-Train Algorithm

Training Data: VAI results (*X*_1_, *X*_2_, …, *X*_N_) and corresponding CoDs (*C*_1_, *C*_2_, …, *C*_*N*_)
Input: VAI result *X*
Output: *C* selected uniformly at random from (*C*_1_, *C*_2_, …, *C*_*N*_)

The confusion matrix for Random-From-Train prediction on the PHMRC validation database adult deaths is shown in Table 4.

**Table 4:** Confusion matrix for Random-From-Train verbal autopsy.

a) Random-From-Train Confusion Matrix				
		Predicted		
		Stroke	Diabetes	Other
True	Stroke	28	14	224
	Diabetes	11	13	122
	Other	223	106	1961

The confusion matrix for Random-From-Train (with a length-three cause-list for clarity). As in Table 2, the entry in each cell counts the number of deaths truly due to the row cause that were predicted to be due to the column cause. This table demonstrates that while Random-From-Train is inaccurate at the individual level, at the population level the prediction distribution can closely match the truth

### Chance-correcting previous results

Although previous work has used an un-chance-corrected version of CSMF accuracy [12, 13, 16, 18, 19, 21, 22], it would be generally useful to have a metric of population-level accuracy where a score of zero indicates predictive accuracy equal to Random Allocation. We therefore set out to correct CSMF accuracy for chance analogously to chance-corrected concordance, and to develop a formula for Chance Corrected CSMF (CCCSMF) accuracy where the quality of random allocation is 0.0, while perfect prediction scores 1.0. To do this, we performed a Monte Carlo calculation of the CSMF accuracy of Random Allocation, by simulating a dataset with known CSMF distribution, assigning “predicted” causes of death uniformly at random, and measuring the CSMF accuracy of the predictions.

The distribution of the simulated dataset is an important and subtle detail of this calculation. We sampled the true CSMF distribution from an uninformative Dirichlet distribution (a probability distribution over CSMFs which gives equal probability to all possible CSMF distributions) [24]. We generated 10,000 replicates of the Monte Carlo simulation, and calculated the mean the CSMF accuracy across all replicates.

We then used the calculated values to chance-correct the CSMF accuracy, according to the formula

$$ CCCSMF=\left( CSMF- mean\  of\  random\  allocation\right)/\left(1- mean\  of\  random\  allocation\right) $$

We also used this simulation framework to perform a Monte Carlo calculation of the concordance for random allocation, which provides a cross-check for the analytical derivation of CCC derived in Murray et al. [13]. We repeated the simulations for cause lists ranging from 3 to 50 causes.

To demonstrate the utility of this view, we updated the comparative performance plot from Murray et al. [12] for all commonly used methods, to use CCCSMF accuracy as the metric of population-level accuracy. This plot compared a range of VA prediction methods in a range of settings according to CCC and CSMF accuracy using a database of VAIs with known underlying cause of death, according to gold-standard clinical diagnostic criteria. As in the previous work, we have presented results for three age groups separately: Adult, Child, and Neonatal deaths (*N* = 7,846, 2,064, and 2,625 respectively). For each age group, in addition to analyzing with all available information, we also excluded all answers to questions that require the deceased to have contact with the health system, such as “Was [name] ever told by a health professional that he or she ever suffered from one of the following?” Following the terminology we developed in our previous work, we call these scenarios with and without healthcare experience (HCE).

This simulation setting also provided us an opportunity to demonstrate the importance of randomly resampling the cause-fraction of the test set from an uninformative Dirichlet distribution (a technical point that perhaps has not been sufficiently appreciated since its introduction in Murray et al. [13]). To do so, we compared the CCCSMF accuracy of Random Allocation with that of Random-From-Train, where training data was either uniformly distributed among causes (as we strongly recommend) or distributed according to the same distribution as the test data (as has sometimes been the case in other work [23]).

We conducted all analysis with Python 2.7 (Additional file 1: Supplementary Text 2).

## Results

**Fig. 1:**
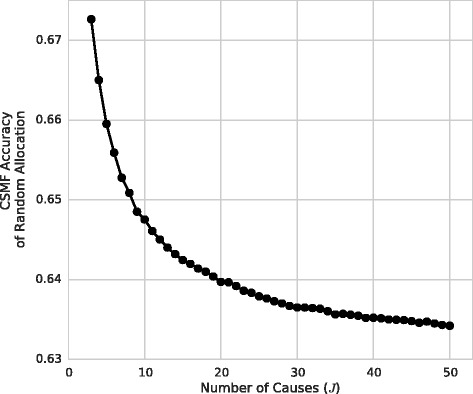
CSMF Accuracy of random allocation as a function of CoD list length. The mean CSMF accuracy of random allocation was calculated with 10,000 Monte Carlo replicates for cause-list length ranging from 3 to 50. The CSMF accuracy decreases monotonically as a function of *J* and appears to stay above 1 − 1/*e* ≈ 0.632, which we selected for our chance-correction parameter

We found that the CSMF Accuracy of Random Allocation decreased slightly and nonlinearly as a function of *J* across the random considered (Fig. 1), and we proved analytically that it tends towards an asymptotic value of 1 − *e*^− 1^ ≈ 0.632 as *J* and *N* tend to ∞ (Additional file 2: Supplementary Text 1). For simplicity, we use this value to produce the same formula for CCCSMF for all values of the CoD list *J* (*J*=6, 21, and 34 are the lengths of the PHMRC cause lists for neonatal, child, and adult deaths [17]):

$$ CCCSMF=\left( CSMF-0.632\right)/\left(1-0.632\right) $$

**Fig. 2:**
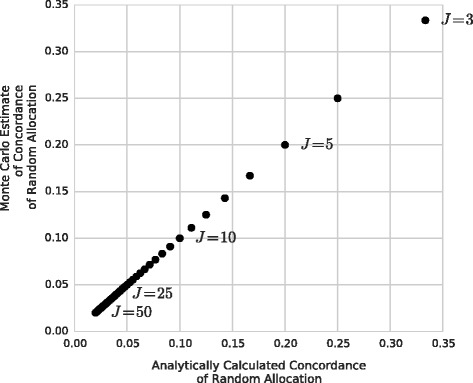
Comparison of concordance from Monte Carlo calculation and analytic calculation. The analogous chance-correction value for concordance was calculated analytically in Murray et al. [13], and we confirmed its accuracy in our simulation environment. The absolute relative difference was always less than 1 %

We used a Monte Carlo estimation procedure to calculate the concordance of random allocation. The results of the estimates agree precisely with the analytical value of 1/*J* used for correcting for change in Murray et al. [13] (Fig. 2, R^2 = 1.0).

**Fig. 3:**
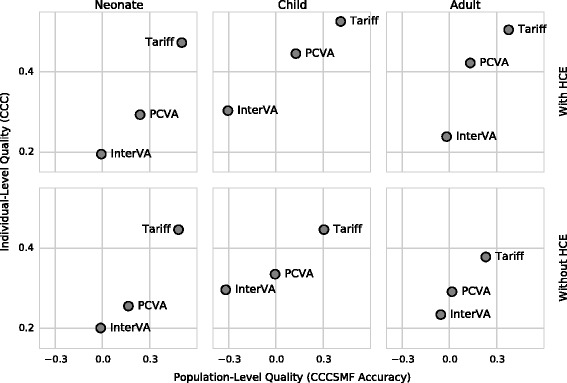
Comparison of individual-level and population-level prediction quality for three commonly used methods: InterVA, Tariff, physician-certified verbal autopsy (PCVA). Questions that rely on the deceased having health care experience (HCE) are necessary for population-level PCVA quality to surpass random guessing. Data from Murray et al. [12]

Using the chance-corrected metrics for the *x*- and *y*-axes, we produced an updated version of the master graphic comparing the individual- and population-level quality of all commonly used VA analysis methods from Murray et al. [12] for neonates, children, and adults, considering and not considering HCE (Fig. 3). For simplicity, we did not include uncertainty quantification, but the same adjustment formula applied to transform the point estimate of CSMF accuracy to CCCSMF accuracy can be used to transform the upper and lower limits of the CSMF accuracy 95 % CI.

When using the Random-From-Train approach with training data drawn from the same CSMF distribution as test data, we measured an unreasonably high CCCSMF Accuracy. Resampling the test set CSMF distribution from an uninformative Dirichlet fixed this problem, and resulted in CCCSMF accuracy for Random-From-Train near zero in a way similar to CCCSMF accuracy of Random Allocation (Table 5).

**Table 5:** CCCSMF accuracy of Random Allocation and Random-From-Train with and without resampling the test CSMF distribution.

*J*	Random-From-Train (Same CSMF)	Random Allocation	Random-From-Train (Resampled CSMF)
5	0.980	0.075	0.092
15	0.964	0.028	0.027
25	0.953	0.016	0.016
35	0.945	0.010	0.007
50	0.933	0.006	−0.005

This table demonstrates the importance of resampling the CSMF distribution in the test set; if the test and train sets have the same CSMF distribution, then simple approaches like Random-From-Train, as well as state-of-the-art approaches like King-Lu [23], can appear to have better performance than is justified, due to “overfitting”

## Discussion

The objective of this study was to develop and test a measure of population-level predictive accuracy that is informative in absolute, as well as relative, terms. We believe that our new metric, Chance-corrected CSMF Accuracy, makes things clearer by increasing the dynamic range of the population-level quality measure; although a method that attains CSMF Accuracy of 0.632 may sound promising in absolute terms, it is not. As shown above, this is the CSMF Accuracy of random guessing. By subtracting 0.632 from the CSMF Accuracy, random guessing and methods of similar quality are given a score near zero. Rescaling the scores by dividing through by 1 - 0.632 maintains the meaningful upper limit of the quality score, where CSMF Accuracy of 1.0 indicates perfect agreement between truth and prediction.

Unlike chance-corrected concordance, CCCSMF Accuracy is *not* essential for comparing different length cause lists. This is because the CSMF Accuracy of Random Allocation is relatively insensitive to changes in cause-list length (it dropped from 0.67 to 0.63 as *J* ranged from 3 to 50 in Fig. 1). This can be compared with the concordance of random allocation for different-length cause lists, which ranged from 0.35 to 0.02 as *J* ranged from 3 to 50 in Fig. 2.

Resampling the CSMF distribution is essential when evaluating CSMF and CCCSMF Accuracy; without it, the trivial approach of Random-From-Train appears to be nearly perfect at the population level. The issue exemplified by Random-From-Train is not merely a theoretical/pathological concern. It has also shown up in practice when evaluating the King-Lu method (a recently developed method for mapping from VAIs directly to CSMFs) [23]. It is likely also relevant in physician-certified verbal autopsy (PCVA), because physicians may rely on their prior beliefs about the composition of disease. Without resampling the test data, a validation method will not be able to contradict a prior belief, even if the belief is incorrect. In other words, just like all machine learning evaluations, it is essential to measure CCCSMF accuracy out-of-sample, but, because CSMF and CCCSMF Accuracy are population-level metrics, measuring out-of-sample predictive validity is more complicated than simply using a train/test split. The PHMRC developed a methodology for this which we recommend [13], and we hope that the demonstration of its importance here will help in its uptake.

### Limitations

Despite the importance of resampling the CSMF distribution of the test set, it is not without limitations. The uninformative Dirichlet assumes that *anything* can happen in test CSMF, because the out-of-sample CSMF is selected uniformly at random. This is the simplest way to address the risk of over-fitting, but it is perhaps too tough a challenge, since there is *some* structure to CSMF distributions that could be assumed.

The VAIs held out for out-of-sample validation were from the same population, selected uniformly at random. This approach may be overly optimistic about performance on VAIs from a completely different population. It would be prudent to replicate validation studies periodically, to guard against differential item functioning and changes symptomology.

The premise that every death has a single, underlying cause has been challenged [25, 26], and as the epidemiological transition continues and more individuals experience multiple comorbidities, this simplifying assumption will become even more tenuous. However, we may still hope to provide meaningful information at the population level.

## Conclusion

Chance-corrected CSMF accuracy is a simple transformation of CSMF accuracy, but we believe that it provides additional clarity on the absolute and relative performance of VA analysis methods at the population level.

The chance-correction of CSMF Accuracy does not change the overall recommendations from Murray et al.: namely that the Tariff 2.0 method is preferred for all applications of automated VA methods [12].

As the epidemiological transition, technology, and costs evolve, the accuracy and cost-effectiveness of alternative approaches to measuring causes of deaths should continue to be assessed. Further innovation will improve the quality of this critical information for decision-making.

## Additional files

Additional file 1:
**Supplementary text 2 Python script.**
Additional file 2:
**Supplementary text 1 Asymptotic calculation.** (PDF 183 kb)

## Abbreviations

CCC: Chance-corrected concordance
CoD: Cause of death
CCCSMF: Chance-corrected cause-specific mortality fraction
CSMF: Cause-specific mortality fraction
CVRS: Civil and vital registration system
HCE: Health care experience
ML: Machine learning
PCVA: Physician certified verbal autopsy
PHMRC: Population Health Metrics Research Consortium
VAI: Verbal autopsy interview
