# Supplementary material for: Measuring causes of death in populations: a new metric that corrects cause-specific mortality fractions for chance
Source: Popul Health Metr. 2015 Oct 12;13:28. doi: 10.1186/s12963-015-0061-1 (PMC4603634; doi:10.1186/s12963-015-0061-1)
Supplement: Additional file 2: — Supplementary text 1 Asymptotic calculation. (PDF 183 kb) [file 12963_2015_61_MOESM2_ESM.pdf]

Supplementary Text 1: Asymptotic calculation of cause-specific mortality accuracy under random allocation

Abraham D. Flaxman

1 Background

This appendix accompanies the paper "Measuring causes of death in populations: a new metric that corrects cause-specific mortality fractions for chance" by Flaxman *et al*, which proposes an approach to chance-correcting the cause-specific mortality fraction (CSMF) accuracy used in measuring the predictive accuracy of verbal autopsy analysis methods at the population level.

In the main paper, the proposed chance-correction formula is based on the CSMF accuracy of the Random Allocation baseline approach, which labels verbal autopsy interviews with an underlying cause of death selected uniformly at random from all causes on a predefined, mutually exclusive, collectively exhaustive, cause list.

The purpose of this appendix is to provide an intuitive justification and rigorous mathematical derivation of the asymptotic CSMF accuracy of Random Allocation, as the length of the cause list, J, and number of examples, N, grow large.

2 Mathematical formulation of the CSMF Accuracy of Random Allocation

The CSMF Accuracy formula from the main text is the following:

$$\text{CSMF Accuracy} = 1 - \frac{\sum_{j=1}^{J} \left|\text{CSMF}_j^{\text{true}} - \text{CSMF}_j^{\text{pred}}\right|}{2\left(1 - \min_j \left(\text{CSMF}_j^{\text{true}}\right)\right)}.$$

This is not mathematically precise, however; as stressed in the main text, it is essential to average CSMF Accuracy over true CSMFs that have been randomly resampled. This can be represented mathematically as E[CSMF Accuracy], where the expectation is taken over: (1) vector $\left(\text{CSMF}_1^{\text{true}}, \text{CSMF}_2^{\text{true}}, \ldots, \text{CSMF}_J^{\text{true}}\right)$ drawn from an uninformative Dirichlet distribution; (2) a test dataset with N examples where the underlying cause follows the CSMF distribution from (1);

and (3) the random choices (if any) in the prediction algorithm used to generate the $\mathrm{CSMF}_j^{\mathrm{pred}}$ values.

In order to calculate the asymptotic value of this expectation, as J and N grow large, it is useful to introduce some additional notation. For fixed J and N, let $X_1, X_2, \ldots, X_J$ be independent, exponentially distributed random variables, with parameter 1, i.e.

$$\Pr[X_j \le x] = (1 - e^{-x})\mathbf{1}[x \ge 0],$$

where $\mathbf{1}[\cdot]$ is an indicator function equal to one if the condition in the square brackets holds, and zero otherwise.

The uninformative Dirichlet distribution on $\left(\mathrm{CSMF}_1^{\mathrm{true}}, \ldots, \mathrm{CSMF}_J^{\mathrm{true}}\right)$ can then be represented by

$$\mathrm{CSMF}_j^{\mathrm{true}} = \frac{X_j}{\sum_{j'=1}^{J} X_{j'}} \text{ for } j = 1, \ldots, J.$$

Under Random Allocation, the predicted CSMFs for different causes are dependent random variables, but in our analysis it will only be necessary to work with the marginal distribution of $\mathrm{CSMF}_j^{\mathrm{pred}}$ for a single j at a time, and this can be expressed in terms of a binomial distribution. Let Y_j be a binomially distributed random variable, with N trials and success probability $1/J$, as defined by the equation

$$\Pr[Y_j = k] = \binom{N}{k} \left(\frac{1}{J}\right)^k \left(1 - \frac{1}{J}\right)^{N-k}.$$

The marginal distribution of $\mathrm{CSMF}_j^{\mathrm{pred}}$ is then given by $\mathrm{CSMF}_j^{\mathrm{pred}} = Y_j/N$.

Putting this notation all together, we have that for J causes and N VAs, the CSMF Accuracy for Random Allocation is

$$\mathrm{E}[\text{CSMF Accuracy}] = \mathrm{E}\left[1 - \frac{\sum_{j=1}^{J} \left| \frac{X_j}{\sum_{j'=1}^{J} X_{j'}} - \frac{Y_j}{N} \right|}{2\left(1 - \min_j \left(\mathrm{CSMF}_j^{\mathrm{true}}\right)\right)}\right].$$

3 Intuitive, but non-rigorous, derivation of the CSMF Accuracy of Random Allocation

With the mathematical formulation of the CSMF Accuracy of Random Allocation from the previous section in hand, it is now possible to provide an intuitively convincing, but mathematically non-rigorous, derivation of its asymptotic value.

First, we note that $\sum_{j=1}^{J} \mathrm{CSMF}_j^{\mathrm{true}} = 1$ and therefore $\min_j \mathrm{CSMF}_j^{\mathrm{true}} \le 1/J$. Since we also have $\mathrm{CSMF}_j^{\mathrm{true}} \ge 0$, the denominator on the right-hand side of

the CSMF Accuracy formula can be approximated by the following when J is large:

$$2\left(1-\min_j \mathrm{CSMF}_j^{\mathrm{true}}\right)\approx 2.$$

Following this approximation, linearity of expectations gives

$$\mathrm{E}[\text{CSMF Accuracy}]\approx 1-\frac{\sum_{j=1}^{J}\mathrm{E}\left[\left|\frac{X_j}{\sum_{j'=1}^{J}X_{j'}}-\frac{Y_j}{N}\right|\right]}{2}$$

Moving our attention now to the expectation on the right-hand side of the above equation, we make two more approximations: For sufficiently large J, the sum $\sum_{j'=1}^{J}X_{j'}\approx J$; and, for sufficiently large N, the random variable $\mathrm{CSMF}_j^{\mathrm{pred}}=Y_j/N\approx\frac{1}{J}$.

These approximations allow us to simplify the expectation in the numerator of the previous approximation to

$$\begin{aligned}\mathrm{E}\left[\left|\frac{X_j}{\sum_{j'=1}^{J}X_{j'}}-\frac{Y_j}{N}\right|\right]&\approx\mathrm{E}\left[\left|\frac{X_j}{J}-\frac{1}{J}\right|\right]\\&=\frac{1}{J}\mathrm{E}\left[|X_j-1|\right].\end{aligned}$$

Following this simplification, the only remaning piece of the puzzle is to calculate the expectation of the absolute value of X_j-1. The following lemma gives the result:

Lemma 3.1. *For X_j an exponentially distributed random variable with parameter 1,*

$$\mathrm{E}\left[|X_j-1|\right]=2/e.$$

We do not prove this here, but it can be shown by the interested reader via two definite integrals.

Putting these pieces together now yields the intended result, approximately:

$$\begin{aligned}\mathrm{E}[\text{CSMF Accuracy}]&\approx 1-\frac{\sum_{j=1}^{J}\frac{1}{J}\mathrm{E}\left[|X_j-1|\right]}{2}\\&=1-\frac{1}{2}\sum_{j=1}^{J}\frac{1}{J}(2/e)\\&=1-1/e.\end{aligned}$$

4 Rigorous derivation

To convert the intuitive approximation in the previous section into a mathematically rigorous proof requires a careful accounting of how just imprecise were the

above approximations. For example, as already mentioned,

$$0 \le \min_j \left(\mathrm{CSMF}_j^{\mathrm{true}}\right) \le 1/J.$$

These rigorous inequalities can be used in place of the first approximate equality of the previous section, yielding

$$\mathrm{E}[\text{CSMF Accuracy}] \ge 1 - \frac{\sum_{j=1}^{J} \mathrm{E}\left[\left|\frac{X_j}{\sum_{j'=1}^{J} X_{j'}} - \frac{Y_j}{N}\right|\right]}{2(1-1/J)};$$

$$\mathrm{E}[\text{CSMF Accuracy}] \le 1 - \frac{\sum_{j=1}^{J} \mathrm{E}\left[\left|\frac{X_j}{\sum_{j'=1}^{J} X_{j'}} - \frac{Y_j}{N}\right|\right]}{2}.$$

To similarly address the other approximations, we now focus our attention on $\mathrm{E}\left[\left|\frac{X_j}{\sum_{j'=1}^{J} X_{j'}} - \frac{Y_j}{N}\right|\right]$ for a specific, fixed j. Our approach will be to define three events that occur with probability suitably close to one that we can calculate or bound the expectation in all cases. Let $\mathcal{A}$ denote the event that Y_j/N is not too far from its mean,

$$\mathcal{A} = \left\{Y_j = N/J \pm 2\log N\sqrt{N}\right\}.$$

Similarly, let $\mathcal{B}$ denote the event that the sum of the $X_{j'}$ variables besides X_j is not too far from its mean,

$$\mathcal{B} = \left\{\sum_{j' \ne j} X_{j'} = (J-1) \pm 2\log J\sqrt{J}\right\}.$$

Finally, let $\mathcal{C}$ denote the event that X_j (which has expected value one, but unbounded support) is not just huge:

$$\mathcal{C} = \{X_j \le 2\log J\}.$$

When $\mathcal{A}, \mathcal{B}$, and $\mathcal{C}$ occur, the complex quantity inside the expectations that bound CSMF Accuracy above can itself be bounded by

$$\begin{aligned}
\left|\frac{X_j}{\sum_{j'=1}^{J} X_{j'}} - \frac{Y_j}{N}\right| &= \left|\frac{X_j}{(J-1) \pm (2\log J\sqrt{J} + 2\log J)} - \frac{N/J \pm 2\log N\sqrt{N}}{N}\right| \\
&= \left|\frac{X_j}{J\left(1 \pm \left(\frac{1}{J} + \frac{2\log J}{\sqrt{J}} + \frac{2\log J}{J}\right)\right)} - \frac{1}{J}\left(1 \pm \frac{2J\log N}{\sqrt{N}}\right)\right| \\
&= \frac{1}{J}\left|X_j - 1\right|\left(1 \pm \mathcal{O}\left(\frac{\log J}{\sqrt{J}} + \frac{J\log N}{\sqrt{N}}\right)\right).
\end{aligned}$$

We now decompose E[CSMF Accuracy] using indicator variables for $\mathcal{A}, \mathcal{B}$, and $\mathcal{C}$, and linearity of expectations:

$$\begin{aligned}
\mathrm{E}\left[\left|\frac{X_j}{\sum_{j'=1}^{J} X_{j'}} - \frac{Y_j}{N}\right|\right] =& \mathrm{E}\left[\left|\frac{X_j}{\sum_{j'=1}^{J} X_{j'}} - \frac{Y_j}{N}\right| \cdot \mathbf{1}[\mathcal{A} \wedge \mathcal{B} \wedge \mathcal{C}]\right] \\
&+ \mathrm{E}\left[\left|\frac{X_j}{\sum_{j'=1}^{J} X_{j'}} - \frac{Y_j}{N}\right| \cdot \mathbf{1}[\neg\mathcal{A} \vee \neg\mathcal{B} \vee \neg\mathcal{C}]\right].
\end{aligned}$$

The first term in this sum can be written using the bound above because $\mathcal{A}, \mathcal{B}$, and $\mathcal{C}$ all occur:

$$\begin{aligned}
&\mathrm{E}\left[\left|\frac{X_j}{\sum_{j'=1}^{J} X_{j'}} - \frac{Y_j}{N}\right| \cdot \mathbf{1}[\mathcal{A} \wedge \mathcal{B} \wedge \mathcal{C}]\right] = \\
&\quad \frac{1}{J}\mathrm{E}\Big[|X_j - 1| \cdot \mathbf{1}[\mathcal{A} \wedge \mathcal{B} \wedge \mathcal{C}]\Big]\left(1 \pm \mathcal{O}\left(\frac{\log J}{\sqrt{J}} + \frac{J \log N}{\sqrt{N}}\right)\right).
\end{aligned}$$

The expectation can now be further simplified, because events $\mathcal{A}$ and $\mathcal{B}$ are independent of X_j:

$$\mathrm{E}\Big[|X_j - 1| \cdot \mathbf{1}[\mathcal{A} \wedge \mathcal{B} \wedge \mathcal{C}]\Big] = \mathrm{E}\Big[|X_j - 1| \cdot \mathbf{1}[\mathcal{C}]\Big]$$

Since the quantity inside the absolute value is at most 1, the second term in the sum above can be bounded by the probability that the events $\mathcal{A}, \mathcal{B}$, and $\mathcal{C}$ do not occur:

$$\begin{aligned}
\mathrm{E}\left[\left|\frac{X_j}{\sum_{j'=1}^{J} X_{j'}} - \frac{Y_j}{N}\right| \cdot \mathbf{1}[\neg\mathcal{A} \vee \neg\mathcal{B} \vee \neg\mathcal{C}]\right] &\le \mathrm{E}\left[\mathbf{1}[\neg\mathcal{A} \vee \neg\mathcal{B} \vee \neg\mathcal{C}]\right] \\
&\le \Pr[\neg\mathcal{A}] + \Pr[\neg\mathcal{B}] + \Pr[\neg\mathcal{C}].
\end{aligned}$$

The ingredients necessary to complete the proof are now the following:

Lemma 4.1. *For X_j an exponentially distributed random variable with parameter 1, and event $\mathcal{C} = \{X_j \le 2\log J\}$,*

$$\mathrm{E}\Big[|X_j - 1| \cdot \mathbf{1}[\mathcal{C}]\Big] = 2/e + \mathcal{O}(1/J).$$

Lemma 4.2. *For Y_j a binomially distributed random variable with parameters n and $1/J$ and event $\mathcal{A} = \left\{Y_j = N/J \pm 2\log N\sqrt{N}\right\}$, we have*

$$\Pr[\neg\mathcal{A}] = \mathcal{O}(1/N).$$

Lemma 4.3. *For $\{X_1, X_2, \ldots, X_J\}$ independent, exponentially distributed random variables each with parameter 1, and event*

$$\mathcal{B} = \left\{\sum_{j' \ne j} X_{j'} = (J-1) \pm 2\log J\sqrt{J}\right\},$$

we have

$$\Pr[\neg\mathcal{B}] = \mathcal{O}(1/\log J).$$

Lemma 4.4. *For X_j an exponentially distributed random variable with parameter 1, and event $\mathcal{C} = \{X_j \le 2\log J\}$, we have*

$$\Pr[\neg\mathcal{C}] = \mathcal{O}(1/J).$$

Once these lemmas are established, substituting them into the bounds above yields a mathematically rigorous analogue of the argument in section 3.

Proof of Lemma 4.1. This proof is a relatively straightforward calculus exercise:

$$\begin{aligned}
\mathrm{E}\Big[\,|X_j - 1| \cdot \mathbf{1}[\mathcal{C}]\Big] &= \int_{x=0}^{\infty} |x-1| \cdot \mathbf{1}[x \le 2\log J] e^{-x} dx \\
&= \int_{x=0}^{1} (1-x)e^{-x} dx + \int_{x=1}^{2\log J} (x-1)e^{-x} dx \\
&= xe^{-x}\Big|_{x=0}^{1} - xe^{-x}\Big|_{x=1}^{2\log J} \\
&= 1/e - 0 - (2(\log J)/J^2 - 1/e) \\
&= 2/e + \mathcal{O}(1/J).
\end{aligned}$$

□

Proof of Lemma 4.2. This follows from a common formulation of Chernoff's inequality (e.g. Corollary 2.3 in [1]):

$$\begin{aligned}
\Pr[\neg\mathcal{A}] &= \Pr\left[|Y_j - N/J| \ge 2(\log N)\sqrt{N}\right] \\
&\le 2\exp\left(-4(\log N)^2 J/3\right) \\
&= \mathcal{O}(1/N),
\end{aligned}$$

provided $N \gg J^2$. □

Proof of Lemma 4.3. This can be shown via Chebyshev's inequality (e.g. equation 1.2 in [1]):

$$\begin{aligned}
\Pr[\neg\mathcal{B}] &= \Pr\left[\left|\sum_{j' \ne j} X_{j'} - (J-1)\right| \ge \sqrt{J}\log J\right] \\
&\le \frac{\mathrm{Var}\left[\sum_{j' \ne j} X_{j'}\right]}{J(\log J)^2} \\
&= \frac{J-1}{J(\log J)^2} \\
&= \mathcal{O}(1/\log J).
\end{aligned}$$

□

Proof of Lemma 4.4. Like the proof of Lemma 4.1, this can be obtained directly from the distribution of X_j using calculus:

$$\begin{aligned}
\Pr[\neg\mathcal{C}] &= \Pr[X_j > 2\log J] \\
&= \int_{x=2\log J}^{\infty} e^{-x} dx \\
&= -e^{-x}\Big|_{x=2\log J}^{\infty} \\
&= 0 - (-e^{-2\log J}) \\
&= J^{-2} \\
&= \mathcal{O}(1/J).
\end{aligned}$$

□

References

[1] S. Janson, T. Luczak, and A. Rucinski, *Random graphs.* John Wiley & Sons, 2011.
